# The PART randomised trial protocol: Partial Ablation versus Radical Treatment for prostate cancer

**DOI:** 10.1111/bju.70448

**Published:** 2026-09-18

**Authors:** Richard J. Bryant, Tom Leslie, Marc Laniado, Abhishek Sharma, Alistair Grey, John Withington, Christopher Blick, Edward Streeter, Sanjeev Kotwal, Oliver Hulson, William Cross, Daniel W. Good, Altan Omer, Taimur T. Shah, Nishant Bedi, Luke Stroman, Caroline Moore, Prasanna Sooriakumaran, Hashim U. Ahmed, Simon F. Brewster, Vicki S. Barber, Aimi Hewitt, Clare Calvert, Jessica Scaife, Derek Shepherd, Filipa Landeiro, Jane Wolstenholme, Philip Camilleri, Clare Verrill, Shelagh Lovell, Martin Pirkl, Fergus Gleeson, Roxanne Williams, Jo Cook, Catherine R. Holt, Martyn Hill, Jonathan Cook, James Radford, Ioana R. Marian, Ruth Macpherson, Daisy Elliott, Jenny L. Donovan, Freddie C. Hamdy

**Affiliations:** ^1^ Department of Urology, Churchill Hospital Oxford University Hospitals NHS Foundation Trust Oxford UK; ^2^ Nuffield Department of Surgical Sciences University of Oxford Oxford UK; ^3^ Surgical Intervention Trials Unit University of Oxford Oxford UK; ^4^ Oxford Clinical Trials Research Unit (OCTRU), Nuffield Department of Orthopaedics, Rheumatology and Musculoskeletal Sciences University of Oxford Oxford UK; ^5^ Patient and Public Involvement Representative, PART Trial Oxford UK; ^6^ Health Economics Research Centre, Nuffield Department of Population Health University of Oxford Oxford UK; ^7^ Department of Oncology, Churchill Hospital Oxford University Hospitals NHS Foundation Trust Oxford UK; ^8^ Department of Cellular Pathology Oxford University Hospitals NHS Foundation Trust, John Radcliffe Hospital Oxford UK; ^9^ Department of Radiology Oxford University Hospitals NHS Foundation Trust, Churchill Hospital Oxford UK; ^10^ Department of Urology, Wexham Park Hospital Frimley Health NHS Foundation Trust Slough UK; ^11^ Harold Hopkins Department of Urology, Royal Berkshire Hospital Royal Berkshire NHS Foundation Trust Reading UK; ^12^ Department of Urology NHS Lothian, Western General Hospital Edinburgh UK; ^13^ Department of Urology University College London Hospitals NHS Foundation Trust London UK; ^14^ Department of Urology, The Royal London Hospital Barts Health NHS Trust London UK; ^15^ Imperial Prostate, Division of Surgery, Department of Surgery and Cancer Imperial College London London UK; ^16^ Imperial Urology, Charing Cross Hospital Imperial College Healthcare NHS Trust London UK; ^17^ Division of Surgery and Interventional Science University College London London UK; ^18^ Department of Urology East Kent Hospitals University NHS Foundation Trust, Kent and Canterbury Hospital Canterbury UK; ^19^ Department of Urology Leeds Teaching Hospitals NHS Trust Leeds UK; ^20^ Department of Urology University Hospitals Coventry and Warwickshire NHS Trust, University Hospital Coventry UK; ^21^ Population Health Sciences University of Bristol Bristol UK; ^22^ NIHR Bristol Biomedical Research Centre, University Hospitals Bristol and Weston NHS Foundation Trust and University of Bristol Bristol UK

**Keywords:** medicine, prostate cancer, prostatectomy, randomised controlled trial, brachytherapy, prostate, quality of life, clinical endpoint, biopsy, prostate brachytherapy

## Abstract

**Objectives:**

Primary objective: to determine whether prostate gland partial ablation (PA) has non‐inferior oncological treatment success vs radical treatment (RT) of localised intermediate‐risk prostate cancer (irPCa). Secondary objectives: to compare (i) participant‐reported urinary and sexual dysfunction side effects, (ii) short/medium term serious adverse events, (iii) health‐related quality of life (HRQoL), (iv) cost‐effectiveness; and to report (v) the need for repeat PA, (vi) accuracy of magnetic resonance imaging and biopsy protocols in determining suitability for PA, (vii) disease progression, local spread and metastases, (viii) medium‐term disease‐specific and overall mortality.

**Participants and Methods:**

The ‘Partial prostate Ablation versus Radical Treatment’ (PART; International Standard Randomised Controlled Trial Number ISRCTN17249875) is a UK‐wide, multicentre, pragmatic, randomised controlled trial investigating whether PA (high‐intensity focused ultrasound [HIFU] or irreversible electroporation [IRE]) is non‐inferior to RT (radical prostatectomy, radical radiotherapy, or low‐dose‐rate brachytherapy [LDR‐B]) in treating localised irPCa. A Qualitative Research Integrated within Trials (QuinteT) Recruitment Intervention is included to optimise recruitment and informed consent. Primary outcome treatment success for PA will be determined by prostate biopsies and imaging, and for RT using standard definitions. Participants will receive treatment according to their randomised allocation, with HIFU or IRE for PA depending on lesion location, and radical prostatectomy, radical radiotherapy, or LDR‐B according to patient and physician preference for RT. Based on 95% and 85% oncological treatment success for RT and PA, respectively, with a non‐inferiority margin of 20%, at median 3 years of follow‐up, PART requires 275 recruited men (137 per group, randomised on a 1:1 basis), for 80% power, with a one‐sided alpha of 0.025. Allowing for a 10% total withdrawal or dropout rate, PART requires 306 recruited and randomised participants.

**Conclusions:**

The PART trial will provide robust prospective data and high‐level randomised evidence to determine oncological treatment effectiveness of PA vs RT for localised irPCa, along with comparative side effect, HRQoL, and health economics evaluations, to inform clinical decision‐making.

**Abbreviations**
ADT: androgen deprivation therapy
bpMRI: biparametric MRI
EPIC: Expanded Prostate Cancer Index Composite
EQ‐5D‐5L: EuroQoL five Dimensions five Levels
HIFU: high intensity focussed ultrasound
HRQoL: health‐related quality of life
IRE: irreversible electroporation
ISUP: International Society of Urological Pathology
LDR‐B: low dose rate brachytherapy
MAX‐PC: Memorial Anxiety Scale for Prostate Cancer
MDT: multidisciplinary team
mpMRI: multiparametric MRI
NICE: National Institute for Health and Care Excellence
NIHR‐HTA: National Institute for Health Research‐Health Technology Assessment
OCTRU: Oxford Clinical Trials Research Unit
PA: partial ablation
PART: Partial prostate Ablation versus Radical Treatment (trial)
(ir)PCa: (intermediate‐risk) prostate cancer
PORPUS‐P: Patient ORientated Prostate Utility Scale
QALY: quality‐adjusted life year
QRI: QuinteT Recruitment Intervention
RCT: randomised controlled trial
RT: radical treatment
TULSA: transurethral ultrasound ablation

## Introduction

Curative treatment for localised intermediate‐risk prostate cancer (irPCa; International Society of Urological Pathology [ISUP] Grade Group 2–3) involves radical treatment (RT) of the whole gland, either with radical prostatectomy, radical radiotherapy (± concomitant androgen deprivation therapy [ADT]), or low‐dose‐rate brachytherapy (LDR‐B) [1]. These treatments have a high cure rate for irPCa [2]; however, whole‐gland RTs have treatment‐related toxicities including adverse effects on urinary function (urinary incontinence and urethral stricture disease), sexual dysfunction, and radiotherapy‐related bowel toxicity [1].

Partial ablation (PA) of the prostate (‘focal therapy’) destroys tumours within the gland [3, 4, 5, 6, 7, 8, 9] using techniques such as high‐intensity focused ultrasound (HIFU), irreversible electroporation (IRE), cryotherapy, vascular‐targeted photodynamic therapy, and transurethral ultrasound ablation (TULSA) [5, 6, 7, 8, 9, 10, 11, 12, 13]. Improved pre‐biopsy PCa imaging using MRI [14, 15] has facilitated PA with more accurate lesion location and needle biopsy diagnosis, and precise tumour destruction with a margin of normal tissue using a PA modality. PA can potentially deliver effective oncological treatment of localised irPCa with a more favourable side effect profile than RT, as suggested from clinical outcomes of large prospective observational cohorts of PA patients [7, 9]. There has been a paucity of large‐scale randomised controlled trials (RCTs) comparing PA vs RT for localised irPCa, and insufficient high‐level evidence for global adoption of PA as standard‐of‐care treatment [16]. Introduction of PA has been undertaken on a registry cohort basis with geographical variation in availability in the UK, with PA being currently available in a small number of expert centres. In the absence of evidence for PA as an effective treatment for irPCa, the National Institute for Health and Care Excellence (NICE) in the UK, and the European Association of Urology (EAU) Clinical Guidelines, state that PA should be delivered within a clinical trial or by special arrangements for clinical governance, consent, audit or research [1, 17, 18].

The UK ‘Partial prostate Ablation versus Radical Treatment’ (PART) feasibility trial demonstrated that it is feasible to recruit participants with irPCa to an RCT of PA vs RP, with most recruited participants accepting their allocated treatment [19, 20]. The UK‐wide multicentre PART RCT builds on the success of the PART feasibility trial, robustly evaluating the performance characteristics of PA vs RT in treatment‐naïve men with localised irPCa. This will inform policymakers, patients, commissioners, guideline bodies, and physicians of the trade‐offs between these treatment options.

## Design and Methods: the PART Trial Protocol

### Overview

The PART trial is a UK‐wide, multicentre (10 centres), RCT assessing the performance characteristics of PA vs RT as treatment for localised irPCa. The aim is to provide policymakers with the requisite high‐quality evidence to establish whether PA is ‘non‐inferior’ to RT in oncological treatment success, and ‘superior’ in side‐effect profile, and could therefore be considered for carefully selected men with irPCa, if oncological outcomes remain within an acceptable margin and patient‐centred outcomes are improved. Primary, secondary and tertiary objectives are listed in Table 1, the trial schema is outlined in Fig. 1.

**Table 1:** Summary of primary, secondary and tertiary objectives of the PART trial.

Objectives	Outcome measures	Time‐point of evaluation of this outcome measure
**Primary objective**		
To determine whether PA provides effective oncological outcomes compared with RT	A non‐inferiority primary outcome analysis of treatment success. Treatment failure defined as: **Post‐PA (HIFU or IRE):** Any of the following: persistent irPCa in a treated area after one repeat PA treatment repeat PA treatment requiring >75% of the prostate to be treated, and/or indication for salvage RT positive biopsies demonstrating high‐risk PCa (ISUP Grade Group 4–5) at any time extraprostatic disease progression defined by imaging, clinical examination, or biopsy findings development of systemic disease requiring long‐term ADT **Post‐radical prostatectomy:** Any of the following: rising serum PSA level reaching ≥0.2 ng/mL following initial reduction to <0.1 ng/mL after radical prostatectomy failure of serum PSA level to fall below 0.1 ng/mL after radical prostatectomy clinical progression to local recurrence/systemic disease the need for long‐term ADT **Post‐radical radiotherapy or post‐LDR‐B** Defined using the RTOG‐ASTRO Phoenix criteria as: a rise of post‐treatment PSA level ≥2.0 ng/mL above the post‐treatment nadir or clinical progression to systemic disease with the need for long‐term ADT	After a minimum of 3 years median follow‐up after treatment, with at least 12 months post‐treatment follow‐up of the last recruited participant
**Secondary objectives**		
To determine whether PA has a reduced side effect profile, and an improved patient‐reported outcomes profile, compared with RT	HRQoL as measured by the PORPUS‐P	Baseline, 6 weeks after treatment, and 3, 6, 9, 12, 15, 18, 24 months after randomisation, thereafter every 12 months until the end of the study
2 To compare the effect on HRQoL of both treatment arms	HRQoL using standard, validated PROMs questionnaires: IPSS, EQ‐5D‐5L, EPIC and MAX‐ PC (modified 18 item)	Baseline, 6 weeks after treatment, and 3, 6, 9, 12, 15, 18, 24 months after randomisation, thereafter every 12 months until the end of the study
3 To compare the effect on health care resource utilisation of both treatment arms	Health care resource utilisation and cost‐effectiveness in terms of cost per QALY	Baseline, 3, 6, 9, 12, 15, 18, 24 months after randomisation, thereafter every 12 months until the end of the study
4 To compare the short and medium term serious adverse events related to treatments in both treatment arms	Short‐, medium‐ and long‐term serious adverse events related to treatments	Short term: 31 days Medium term: 3 years
5 To determine the proportion of patients needing repeat PA treatment	Proportion of patients needing repeat PA treatment in the PA group	Documentation of the need for repeat PA treatment in the PA group – at 1 year and 3 years after treatment (for those that reach this time point in the study's lifetime)
6 To evaluate the accuracy of current mpMRI/bpMRI imaging and biopsy protocols in determining suitability of patients for PA	The proportion of radical prostatectomy patients who have high‐risk ISUP Grade Group 4–5 disease or bilateral/multifocal ISUP Grade Group 2–3 disease	After histopathological evaluation (only in men who have had radical prostatectomy)
7 To determine disease progression beyond the prostate, including local spread and development of metastases	Time to disease progression (including local and distal recurrence)	At a minimum 12 months after treatment follow‐up of the last treated participant but will be longer for those recruited and treated earlier in the study
8 To determine medium‐term disease‐specific and overall mortality	Time to disease‐specific and overall mortality	At a minimum 12 months after treatment follow‐up of the last treated participant but will be longer for those recruited and treated earlier in the study
**Tertiary objectives (subject to additional funding)**		
To compare the long‐term serious adverse events related to treatments in both treatment arms	Long‐term serious adverse events related to treatments	Up to 15 years after randomisation
2 To determine disease progression beyond the prostate, including local spread and development of metastases	Time to disease progression (including local and distal recurrence)	Up to 15 years after randomisation
3 To determine long‐term disease‐specific and overall mortality	Time to disease‐specific and overall mortality	Up to 15 years after randomisation

ASTRO, American Society for Therapeutic Radiology and Oncology; PROM, patient‐reported outcome measure; RTOG, Radiation Therapy Oncology Group.

**Fig. 1:**
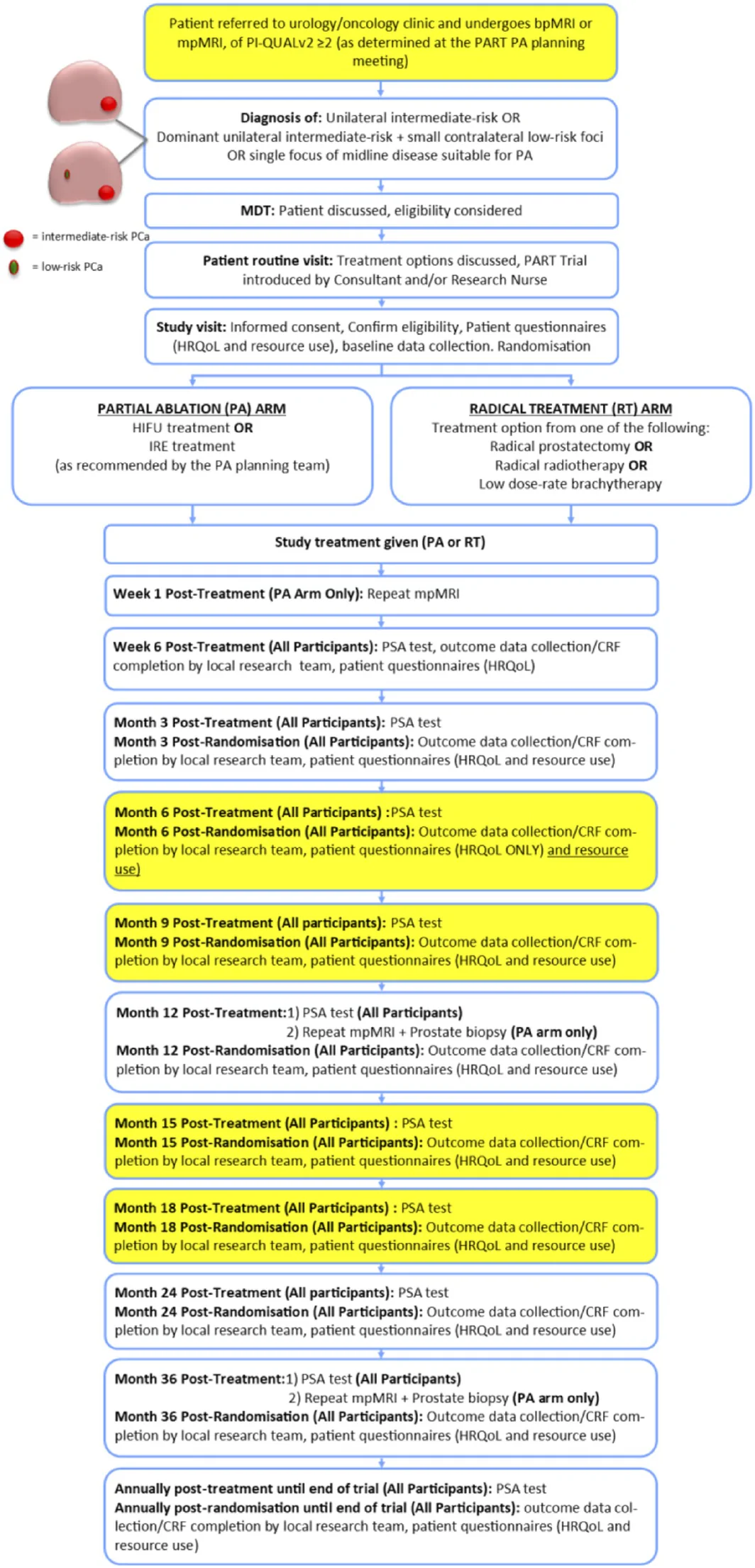
Trial schema. CRF, case report form; PI‐QUAL v2, Prostate Imaging Quality version 2.

### Regulatory Approvals

The PART trial (International Standard Randomised Controlled Trial Number ISRCTN17249875) was approved (Ethics Ref:23/SC/0012) by the Oxford B Research Ethics Committee, is funded by the UK National Institute for Health Research‐Health Technology Assessment programme (NIHR‐HTA; funder reference 17/150/01), and is conducted within the registered UKCRC Oxford Clinical Trials Research Unit (OCTRU) at the University of Oxford following their Standard Operating Procedures, ensuring compliance with principles of International Council for Harmonisation Good Clinical Practice, and is managed by the Surgical Intervention Trials Unit. A data safety and monitoring committee has been appointed to independently review data on safety, protocol adherence, and recruitment in accordance with the Data Monitoring Committees: Lessons, Ethics, Statistics (DAMOCLES) charter. A Trial Steering Committee with independent members provides study supervision on behalf of the funder.

### Study Population, and Inclusion/Exclusion Criteria

The PART trial aims to recruit 306 treatment‐naïve men with localised irPCa suitable for PA (HIFU or IRE based on MRI‐visible tumour location, with concordant diagnostic histology), following good quality (i.e., Prostate Imaging Quality [PI‐QUAL] version 2 score ≥2) pre‐biopsy biparametric MRI (bpMRI) or multiparametric MRI (mpMRI) within the previous 6 months as reviewed at a PART PA multidisciplinary team (MDT) meeting, and either TRUS‐guided or local anaesthetic transperineal biopsy, including targeted and systematic cores. Participants may also be recruited following active surveillance. Inclusion and exclusion criteria are listed in Table 2.

**Table 2:** Inclusion and exclusion criteria for the PART trial.

Inclusion criteria
A patient will be eligible for inclusion in this study if all of the following criteria apply:
Age ≥18 years with unilateral clinically significant irPCa ISUP Grade Group 2–3, or dominant unilateral clinically significant irPCa ISUP Grade Group 2–3 and contralateral ISUP Grade Group 1 (3 + 3) PCa, or with a midline irPCa ISUP Grade Group 2–3 amenable to a PA treatment
2 PSA level ≤20 ng/mL within the last 120 days
3 Pre‐biopsy bpMRI or mpMRI, of adequate diagnostic quality as determined at the PART PA MDT, within the previous 6 months, and bilateral biopsies of the prostate (transrectal or transperineal, and targeted biopsy for visible lesions)
4 Clinically ≤T2c irPCa ISUP Grade Group 2–3 judged by results of DRE, imaging by MRI and biopsy (ISUP Grade Group 1 lesions on the side contralateral to the irPCa are acceptable; a midline single focus of clinically significant ISUP Grade Group 2–3 irPCa, if deemed suitable for PA, is also acceptable)
5 Fit, eligible, with a standard‐of‐care recommendation for any or all of radical prostatectomy, radical radiotherapy or LDR‐B, and suitable for PA using at least one of IRE or HIFU
6 An understanding of the English language sufficient to receive written and verbal information about the study, its consent process and complete study questionnaires
Exclusion criteria
A patient will not be eligible for the study if ANY of the following apply:
Taking part in another therapeutic prostate cancer clinical trial or has been involved in such trials within the previous 4 months
2 PSA level >20 ng/mL within the last 120 days
3 Unfit for RT or general anaesthesia or cannot tolerate TRUS
4 In the opinion of the treating physician, has a contraindication to either HIFU or IRE
5 Not suitable for MRI or has a single or bilateral hip replacement
6 Has evidence of extraprostatic extension by MRI, or clinical or radiological ≥T3 disease
7 Concomitant cancer or previous active treatment for PCa
8 Evidence of metastatic disease
9 Bilateral foci of intermediate‐risk disease or higher
10 Low‐risk (ISUP Grade Group 1) disease only, or high‐risk (ISUP Grade Group ≥4) PCa only
11 History of spontaneous unprecipitated acute urinary retention within the last 6 months prior to entry to the study (a precipitated acute urinary retention episode, e.g., secondary to previous prostate biopsy or UTI, is acceptable)
12 Prostatic calcification and cysts causing ultrasonic shadowing of >1 cm
13 History (within 3 years) of inflammatory bowel disease or any condition that may increase the risk of recto‐urethral fistula formation
14 Has known hypersensitivity to pancuronium bromide, atricurium or cisatricurium, or any medical condition such that muscle relaxation cannot be administered as part of a general anaesthetic
15 Has a history of bladder neck contracture
16 Had active treatment for a malignancy within 3 years, including malignant melanoma, except other types of skin cancer
17 Has any active implanted electronic device incompatible with either MRI or IRE (e.g., pacemaker) (N.B. some newer devices may be compatible with MRI and/or IRE – if compatible, that is acceptable)
18 Is unable or unwilling to be catheterised
19 Has had prior or current PCa therapies
20 Has had prior TURP, urethral stricture surgery, urethral stent or prostatic implants
21 Has had prior major rectal surgery (except for haemorrhoids)
22 Is actively bleeding, is anticoagulated or on blood thinning medications that cannot be stopped for the perioperative period for a PA procedure or has a significant bleeding disorder that may affect the perioperative period as judged by the clinical staff.
23 Unable to give consent to participate in the trial as judged by the clinical staff
24 Wishing to maintain future fertility

### Outcome Measures and End Points

The primary outcome is successful irPCa treatment at the 3‐year median clinical follow‐up (Table 1). Treatment failure after PA is presence of any of: (a) persistent irPCa in a treated area after one repeat PA, (b) repeat PA requiring treatment of >75% of the gland, and/or indication for salvage RT, (c) biopsies containing high‐risk PCa (ISUP Grade Group 4–5), (d) extraprostatic disease progression, (e) systemic disease requiring long‐term ADT. For RT, treatment failure after radical prostatectomy is any of: (a) rising PSA level reaching ≥0.2 ng/mL following initial reduction to <0.1 ng/mL, (b) failure of PSA level to fall <0.1 ng/mL, (c) local recurrence/systemic disease, (d) need for long‐term ADT; treatment failure after radical radiotherapy or LDR‐B is any of: (a) rise of post‐treatment PSA level ≥2.0 ng/mL above the post‐treatment nadir, or (b) extraprostatic and/or systemic disease needing long‐term ADT. Because PSA kinetics, residual prostate tissue and the methods of local assessment differ after PA vs RT, the PART trial applies arm‐specific failure criteria that together define the single clinical construct of failure of the allocated curative‐intent treatment to control localised irPCa.

Secondary outcomes include: rates of patient‐reported side effects of treatment (using the Patient ORientated Prostate Utility Scale [PORPUS‐P]); HRQoL (using standard, validated questionnaires: IPSS; EuroQoL five Dimensions five Levels [EQ‐5D‐5L]; Expanded Prostate Cancer Index Composite [EPIC]; and Memorial Anxiety Scale for Prostate Cancer [MAX‐PC]); cost effectiveness (based on health care resource utilisation and cost‐effectiveness in terms of cost per quality‐adjusted life year [QALY]); short/medium term serious adverse events related to treatments; the proportion of PA patients needing a repeat PA treatment; ability of mpMRI/bpMRI and biopsy protocols to determine patient suitability for PA (based on the proportion of radical prostatectomy patients with ISUP Grade Group 4–5 disease, or bilateral/multifocal ISUP Grade Group 2–3 disease, on histology of the surgical specimen); disease progression locally or development of metastases; medium‐term disease‐specific and overall mortality (Table 1).

### Study Stages

The PART trial had a successful internal pilot phase, which evaluated willingness of men to consent to recruitment and randomisation. The full trial has continued and will include results of men recruited in the pilot.

### Screening

Centres will identify potential participants through diagnostic pathways and cancer MDT meetings, and the research team will screen potentially eligible participants (Table 2). Informed consent for participation will be obtained either remotely and electronically via e‐mail and a telephone call, or in person in clinic. Prior to recruitment and randomisation of each participant the MRI imaging and most recent prostate biopsy result will be reviewed at a PART PA MDT to ensure suitability for PA (HIFU or IRE).

### Randomisation

Consented participants will be randomised to receive either PA or RT using a centralised validated computer randomisation programme through a secure encrypted web‐based service (RRAMP, https://rramp.octru.ox.ac.uk), provided by OCTRU, accessed via the study's Research Electronic Data Capture (REDCap) database. Randomisation will be performed using a minimisation algorithm (or randomisation schedules) to ensure balance between treatment groups using minimisation factors: (a) Age group (<65, 65–75, and >75 years); (b) Baseline ISUP Grade Group (2 or 3); (c) PSA level (<10 and 10 to ≤20 ng/mL); (d) whether the disease is entirely unilateral clinically significant irPCa, or dominant unilateral clinically significant irPCa with contralateral ISUP Grade Group 1 disease. The first few participants will be randomised using a simple randomisation schedule, prepared by the trial statistician, to seed the minimisation algorithm, and a non‐deterministic probabilistic element will be included to prevent predictability of treatment allocation.

### Blinding

Participants will be informed of their treatment allocation immediately after randomisation as it is not possible to blind due to nature of the intervention. The trial statistician, health economist, data safety monitoring committee, clinical team, database programmers, data management and data entry personnel will not be blinded to the treatment allocation. It is not possible to blind the Principal Investigators as they may be the patient's primary physician, and in instances where serious adverse events are reported the Chief Investigator will also be unblinded for full causality assessment. The Trial Steering Committee will remain blinded during review of accruing data and safety.

### Recruitment and Treatment Sites

As with the PART feasibility trial [19, 20], an integrated Qualitative Research Integrated within Trials (QuinteT) Recruitment Intervention (QRI) is included to identify and overcome recruitment issues. PART QRI involves analysis of screening and recruitment data, interviews with trial staff, and audio‐recordings of recruitment discussions, with findings used iteratively to provide tailored feedback and training to optimise recruitment and informed consent.

Recruiting centres in the PART trial need to offer RT ± PA to patients with irPCa. PA does not need to take place in each recruiting centre; if the PA treatment recommended by the PART PA MDT is not available within the recruiting centre, the participant will be referred to an appropriate nearby PART trial centre delivering this PA therapy.

### Partial Ablation

Participants randomised to PA will undergo IRE or HIFU. The PART PA MDT meetings (or, where it exists at sites, a Focal Therapy MDT), attended by, as a minimum, a physician who administers PA, along with a uro‐radiologist, will guide recommendations regarding whether HIFU or IRE may be used on a case‐by‐case basis, based on prostate size, tumour location, and biopsy.

The HIFU will be performed using either the Focal One® (EDAP TMS, Austin, TX, USA) or Sonablate® device (Sonacare Medical, Charlotte, NC, USA). Both devices are Conformité Européenne (CE) marked and will be used in accordance with their indicated use. The device used will be determined by local availability. Trained HIFU physicians will carry out the procedure on PART trial participants. Physicians without HIFU experience will only perform treatments in the presence of a proctor/mentor.

The IRE will be performed using the NanoKnife® medical device (manufactured by Angiodynamics, Latham, NY, USA), using the same principles as above for HIFU.

### Repeat HIFU or IRE Treatments

Repeat PA treatment will be considered, based on repeat imaging and biopsies, if: (i) irPCa suitable for PA on the contralateral side to the original treated malignancy; and/or (ii) irPCa in the previously treated (ipsilateral) side. Each participant may have one repeat PA treatment, which may use a different modality (HIFU or IRE) provided less than 75% of the prostate is treated cumulatively. PA will have failed if there is irPCa in the same region of the prostate gland following repeat treatment.

### Radical Treatment (Standard of Care and Comparator)

Participants randomised to RT will receive one of radical prostatectomy, radiotherapy, or LDR‐B, following discussion with their local urologist and oncologist, through shared decision‐making, delivered by expert specialists.

### Training

Training and proctoring may be required for some physicians delivering HIFU or IRE and will be undertaken by physicians with PA expertise. The ability to independently deliver PA treatment to PART trial participants will be based on competency, with an expert proctor signing off PART physicians in HIFU/IRE based on demonstrable competency following proctoring/training within the PART trial. Training and support for staff recruiting participants will be provided by QRI staff for individuals, sites, and at overall investigator meetings, to optimise recruitment.

### Collection of Outcome Data and Follow‐up Assessments

Participants will receive research specific visits and communications from the study team to collect outcome data. Follow‐up will take place at 6 weeks after treatment, and 3‐monthly after treatment in the first year, then every 6 months for up to 5 years after randomisation depending on recruitment date (Table 3). If, at any point, disease progression is suspected, the participant's cancer will be re‐staged. Participants undergoing PA will receive mpMRI at 1 week, 12 months and 36 months after treatment, and systematic ± targeted (depending on mpMRI result) prostate biopsies at 12 months and 36 months after treatment. For participants who have undergone PA, additional imaging and repeat biopsies may also be triggered by a rise in PSA level of ≥50% over a period of 12 months, or due to any clinical concern. Participants will be sent HRQoL questionnaires (PORPUS‐P, IPSS, EPIC, EQ‐5D‐5L, and modified 18‐item MAX‐PC), and health care use questionnaires at 6‐weeks after treatment, and at 3, 6, 9, 12, 15, 18, 24 and 36 months after randomisation, and every 12 months thereafter until end of study. Treatment complications and unexpected, serious adverse events related to the study intervention will be collected using a combination of medical notes review, Health Care Use Questionnaire review and asking participants at follow‐up appointments, and/or when a member of the research team becomes aware that a serious adverse event has occurred.

**Table 3:** Summary of follow‐up evaluations in the PART trial.

	Day/Weeks (W)/ Months (M) after randomisation	Screening, consent and eligibility	Baseline pre‐ treatment	Treatment start§	Day 7	6 W ± 2 W§	3 M ± 4 W	6 M ± 4 W	9 M ± 4 W	12 M ± 4 W	15 M ± 4 W	18 M ± 4 W	24 M^‡^ ± 4 W	36 M ± 4 W	Annually until study ends
	Initial eligibility screen	X													
	Consent for ‘Information Study’^†^	X													
	QRI audio‐recordings of study discussions with potential participants	X													
	Informed consent for main PART trial		X												
	Eligibility assessment		X												
	PSA blood test§		X^*^			X	X	X	X	X	X	X	X	X	X
	Randomisation		X												
	Baseline data collection/CRF completion		X												
RT ARM ONLY	Choose RT (radical prostatectomy, radical radiotherapy, or LDR‐B)		X												
	RT			X											
PA ARM ONLY	Choose PA modality (HIFU or IRE)		X												
	PA treatment			X											
	mpMRI§				X					X				X	
	Prostate biopsy§									X				X	
	Repeat PA treatment	Repeat PA upon prostate biopsy showing irPCa													
	Outcome data collection/CRF completion by local research team		X	X		X	X	X	X	X			X	X	X
		Safety reporting will continue throughout													
	HRQoL questionnaires		X			X	X	X		X	X	X	X	X	X
	Health care use questionnaire		X				X	X		X	X	X	X	X	X

* Assessments are from start of treatment; all other follow‐up time points are after randomisation.

^†^ Assessments will be performed annually to end of study.

^‡^ OPTIONAL consent.

^§^ Blood test for PSA will be performed to confirm eligibility if no PSA test has been taken in the previous 120 days.

CRF, case report form.

Surveillance after PA is more intensive than after RT because untreated prostate tissue remains *in situ*. Any residual or metachronous disease is therefore more likely to be detected after PA, and detected earlier than following RT. The primary outcome will be interpreted alongside the rates of salvage treatment, extraprostatic progression, development of metastases, and use of systemic treatment, which are less influenced by how intensively each arm is monitored.

All data will be processed according to the General Data Protection Regulation and Data Protection Act 2018, and all documents will be stored safely in confidential conditions.

### Health Economics Analysis

A health economic evaluation will assess the health care resource utilisation, cost impact and cost‐effectiveness in terms of cost per QALY of treating patients with irPCa using PA compared with RT. Using an NHS perspective for the baseline analysis, data will be collected on health care resource use via a questionnaire to be completed by participants. Costs will be estimated using a standardised approach. Data will also be collected on HRQoL using the EQ‐5D‐5L questionnaire. A ‘within‐trial’ cost‐utility analysis will be conducted comparing the implementation of PA vs RT for participants with irPCa.

### Patient Impact

The PART trial will provide accurate and comparative information regarding the trade‐offs in short‐, medium‐ and long‐term oncological efficacy and treatment‐related side effects for PA vs RT for future patients diagnosed with irPCa. It will also provide clinical guidelines and policymaker committees with the necessary evidence to determine whether PA should be adopted as a standard‐of‐care treatment option for patients with localised irPCa.

### Sample Size

Available data indicate that ~5% of men with irPCa undergoing radical prostatectomy or radical radiotherapy demonstrate treatment failure (i.e., clinical or biochemical relapse) within a median of 3 years of follow‐up, with most failures occurring in the first 3 years after treatment, therefore treatment success after RT for localised irPCa is expected to be ~95%. Evidence from observational cohort studies [7] suggests that the oncological success rate for PA at a median 3‐year follow‐up may be somewhat lower (treatment success rate of 85%, i.e., 15% failure rate); however, there has to date not been a large scale RCT directly comparing the oncological efficacy of PA vs RT, and definitions and rates of treatment failure vary. It is therefore possible that PA may have a slightly lower oncological efficacy and treatment success rate compared to RT. It is also hypothesised that PA is superior to RT in terms of functional outcomes. The PART trial is therefore a non‐inferiority oncological outcome trial, with a primary outcome of treatment (i.e., oncological) success. This has been chosen as the primary outcome as each of the PA and RT are delivered with curative intent for localised irPCa. The sample size is driven by the assumptions of 95% (RT) and 85% (PA) oncological treatment success, with a non‐inferiority margin of 20%, at a median 3 years of follow‐up (binary outcome). This requires 275 recruited men (137/138 per group, randomised on a 1:1 basis), for 80% power, with a one‐sided alpha of 0.025. Allowing for a 10% total withdrawal or dropout rate across the entire study, the PART RCT requires 306 men to be recruited and randomised.

The 20% non‐inferiority margin represents the largest difference in treatment success that would be regarded as clinically acceptable. This recognises that PA preserves a wider range of salvage options at lower morbidity than salvage therapy after failed whole‐gland RT. A patient who fails PA can still be offered repeat PA, or salvage radical prostatectomy, radiotherapy, or brachytherapy. Conversely, the salvage options after radical prostatectomy or radical radiotherapy are more limited and carry greater functional cost. The 20% margin applies only to treatment success; any difference of this magnitude in metastasis‐free or cancer‐specific survival, which are reported as secondary outcomes, would be clinically unacceptable. The primary outcome will therefore be interpreted together with the functional, quality of life, re‐treatment, salvage, adverse‐event, and health‐economic outcomes.

### Statistical Analysis

The proportion of treatment success will be compared between RT and PA using a one‐sided 95% CI (Newcombe's method 10 confidence model) [21] to assess inclusion of a non‐inferiority margin of 0.2 (i.e., PA 20% lower than RT) using available follow‐up. Sensitivity analyses will restrict data to 1, 2 and 3 years minimum respectively to assess impact of varying lengths of clinical follow‐up. Survival analysis techniques will also be used in secondary analyses using log rank test, and Kaplan–Meier survival curves will be used for graphical representation. Patients without treatment failure will be censored at time of death or time of last follow‐up. Additional supporting analyses will be undertaken using Cox proportional hazards regression models adjusting for minimisation factors and time from randomisation to start of treatment. For PORPUS‐P and the other disease‐specific HRQoL repeated‐measure outcomes, mixed models will be used to evaluate differences over time by treatment group. These models will be adjusted for treatment, baseline score, minimisation factors, visit, and a treatment by visit interaction as fixed‐effect terms, with patient included as a random effect. Differences between treatment groups will be reported as mean difference with 95% CIs. HRQoL forms that are returned partially complete will be analysed according to the scoring instructions for each questionnaire. The mixed‐model analyses will minimise the impact of missing data under a ‘missing at random’ assumption, therefore the use of multiple imputation or other methods for dealing with missing data is not planned for secondary outcomes. Survival analysis techniques, as described for the treatment success (time to failure) primary outcome and secondary outcomes, will also be used to compare differences between PA and RT treatment for time to disease progression (including metastasis), time to disease‐specific mortality, and time to all‐cause mortality.

The principal analyses are planned at the point where median follow‐up (from randomisation) will reach 3 years. A one‐sided 95% CI for the difference in treatment success will be calculated and assessed against the presence or absence of 20% or more in favour of RT. PA will be considered non‐inferior if the 95% CI does not include a 20% margin in favour of RT, with the primary outcome being analysed on both an intention‐to‐treat and a per‐protocol basis, with the two analyses reported alongside each other when non‐inferiority is judged, and with non‐inferiority only being concluded if it is supported by both analyses. A two‐sided *P* ≤ 0.05 will be considered statistically significant for secondary outcomes. The 95% CIs of comparative treatment effects will be reported. Under the main analysis participants’ data will be analysed as they were randomised irrespective of the treatment received, provided they received one of the trial treatment options (PA or RT). The main analysis set will include all men who have given their informed consent. Secondary analyses will focus on those who complied with per‐protocol treatment allocation. A statistical analysis plan with full details of all analyses planned for the study data will be drafted early in the trial and finalised prior to any primary outcome analysis.

## Results

The PART trial commenced recruitment on 1 April 2023 and successfully transitioned through its internal pilot phase. The main trial is now ongoing. No formal interim analyses of treatment effects are planned for any of the study outcomes. The end of trial results will be reported following the CONsolidated Standards Of Reporting Trials (CONSORT) guidelines.

## Discussion

There is much interest among patients and urologists for PA to become widely available as a standard‐of‐care treatment option for localised irPCa, driven by the desire to reduce treatment‐related side effects of RT. However, to date there is an absence of evidence regarding the trade‐offs between oncological efficacy and treatment‐related side effects for PA vs RT to support making PA a standard‐of‐care therapy option. Several observational cohort series have been published describing PA outcomes up to a median follow‐up of 7 years [7], including using propensity score matching [22, 23]. Whole‐gland RT has to date been considered the established standard‐of‐care treatment for localised irPCa; however, if PA was to be demonstrated to be ‘non‐inferior’ in oncological efficacy compared to RT, then this evidence would be important for patients, physicians, and clinical guidelines committees when evaluating the role of PA vs RT. HRQoL, and urinary and sexual function, and other functional outcomes, are important secondary outcomes in the PART trial, with PA hypothesised to have better HRQoL and functional outcomes compared with RT. The PART trial will provide important, randomised data regarding whether PA results in better HRQoL and functional outcomes compared with RT, and in what ways, as reported by patients themselves. This evidence will be crucial in the consideration of the trade‐offs of PA vs RT, with patients potentially willing to trade small detriments in cancer control for rapid return to normal activities and maintenance of continence and sexual function after treatment [24].

In the UK to date PA has only been available in a small number of centres. However, interest in PA as a standard‐of‐care treatment option for localised irPCa is growing globally. This interest is likely to increase given increasing opportunistic PSA testing and the drive towards population‐based screening, which will inevitably increase the number of patients diagnosed with localised irPCa each year, many of whom will seek and/or be offered treatment.

There are several other ongoing clinical trials in the field. The FARP trial (NCT03668652) [25, 26] from Norway investigating focal ablation (using HIFU or TULSA) vs radical prostatectomy for unilateral ISUP Grade Group 2–3 PCa has reported preliminary findings [26] demonstrating that at the 3‐year follow‐up the rate of treatment failure in the focal ablation (PA) group was non‐inferior to that in the radical prostatectomy group (seven of 107 [6.5%] in the focal ablation group [95% CI 3–13%], vs nine of 106 [8.5%] in radical prostatectomy group [95% CI 5–15%], resulting in a difference of 2% [95% CI –6 to 8%]). The PRIS trial (NCT05513443) [27] in Sweden is investigating IRE vs radical prostatectomy or radical radiotherapy and aims to recruit 184 patients with newly diagnosed irPCa, with a functional (urinary incontinence and irritative symptoms) primary outcome. The ENFORCE trial (NCT06223295) [28] in The Netherlands is investigating focal therapy (using TULSA, IRE or HIFU) vs usual care RT (radical prostatectomy or radical radiotherapy), and aims to recruit 356 participants with localised irPCa, with a primary outcome of oncological non‐inferiority and secondary outcomes of QoL. In France, the medico‐economic EMERHIT study (NCT05710861) [29] is investigating focal HIFU vs radical prostatectomy and aims to recruit 348 participants with irPCa to estimate the cost and utility of PA vs radical prostatectomy from the health system perspective.

The PART trial commenced recruitment in April 2023 and, supported by an integrated QRI, aims to complete accrual by end of 2027, reporting findings in 2029/2030. The study has a pragmatic design and aims to provide real‐world level‐one randomised evidence of oncological treatment effectiveness, and functional and HRQoL side effects, and a full health economics analysis, of PA vs RT for localised irPCa, to inform patients, physicians, and policymakers, ahead of potential guidelines statements from NICE and others. It will contribute significantly to clinical guidance in this increasingly common area of urological practice. The PART trial has the potential to be one of the practice‐defining RCTs regarding modern‐day PA treatment for localised irPCa.

## Disclosure of Interests

Richard J. Bryant receives research grant support from NIHR and Prostate Cancer UK and has previously received a travel grant to present at the FOCAL+ Annual Meeting (National Harbour, USA, 2026). Hashim U. Ahmed receives infrastructure support from the NIHR Imperial Biomedical Research Centre and NIHR/Cancer Research UK (CRUK) Imperial Experimental Cancer Medicine Centre, the Wellcome Trust, the UK NIHR, the UK Medical Research Council, CRUK, Prostate Cancer UK, The Urology Foundation, the British Medical Association Foundation, Imperial Health Charity, and Imperial Prostate Foundation. He was awarded NIHR Senior Investigator status in 2023. Hashim U. Ahmed has previously received travel grants and paid proctorships and trial grants from Sonablate; was a paid consultant for Sophiris Biocorp; sponsored conference attendance by Angiodynamics; medical advisory board for Janssen previously in the last 3 years; is a proctor for Rezum treatment and cryotherapy for Boston Scientific and a paid proctor for HIFU by Sonablate. Hashim U. Ahmed has received paid speaker honorariums from Varian Medical and Angiodynamics.

## Data Availability

Data sharing not applicable to this article as no datasets were generated or analysed during the current study.
